# Language Validation and Cultural Adaptation of the Italian Version of the Family Caregiver Quality of Life Scale

**DOI:** 10.3390/nursrep14030171

**Published:** 2024-09-06

**Authors:** Simone Debenedetti, Simone Cosmai, Daniela Cattani, Stefano Mancin, Giovanni Cangelosi, Fabio Petrelli, Beatrice Mazzoleni

**Affiliations:** 1 Cardiology Unit, Ospedale Maggiore della Carità, Via Largo Bellini, 28100 Novara, Italy; simone.debenedetti94@gmail.com; 2 Department of Biomedical Sciences, Humanitas University, Via Rita Levi Montalcini 4, Pieve Emanuele, 20072 Milan, Italy; simone.cosmai@hunimed.eu (S.C.); daniela.cattani@humanitas.it (D.C.); beatrice.mazzoleni@hunimed.eu (B.M.); 3 Cancer Center, IRCCS Humanitas Research Hospital, Rozzano, 20089 Milan, Italy; stefano.mancin@humanitas.it; 4 Units of Diabetology, ASUR Marche, 63900 Fermo, Italy; 5 School of Medicinal and Health Products Sciences, University of Camerino, 62032 Camerino, Italy; fabio.petrelli@unicam.it

**Keywords:** heart failure, quality of life, caregiver, linguistic validation, nursing assessment scale

## Abstract

Background: Heart failure significantly impacts healthcare systems and society, affecting quality of life (QoL) due to its symptoms and continuous care needs. Nurses are crucial in managing heart failure, supporting both patients and caregivers who face physical, emotional, social, and spiritual challenges. The Family Caregiver Quality of Life (FAMQOL) scale evaluates caregivers’ QoL across all dimensions. This study aims to translate and culturally adapt the FAMQOL from English to Italian, enhancing its utility in nursing research and practice to better identify and support caregiver well-being. Methods: Following EORTC guidelines (2017), the FAMQOL underwent linguistic validation and cultural adaptation. This included independent forward translations from English to Italian, back translations, and reconciliation discussions to produce a testable translation. A pilot test with 15 caregivers assessed the questionnaire’s acceptability and comprehensibility. Results: Linguistic adjustments ensured the questionnaire’s understandability in Italian. Interviews confirmed its acceptability and comprehensibility, with minor modifications enhancing clarity. Conclusions: The translation process successfully adapted the FAMQOL for Italian caregivers. This tool is essential for nursing research and practice, providing a culturally relevant assessment of the burden of care. It allows targeted interventions to support health workers, intercepting the QoL of caregivers early and, consequently, the well-being of patients with heart failure.

## 1. Introduction

Heart failure is one of the most prevalent diseases of the cardiovascular system globally, defined as a “clinical syndrome with signs and/or symptoms caused by a structural and/or functional cardiac abnormality” [1]. In the current context, heart failure is considered a steadily increasing pandemic disease and one of the chronic conditions with the highest social and economic cost, with a prevalence of approximately 63 million cases globally and a burden at the European level that is between USD 5000 and USD 18,000 annually per patient [2,3]. The symptoms most commonly reported by patients with heart failure include dyspnea, chest pain, and asthenia. They negatively affect the patient’s autonomy and quality of life [4]. Quality of life is defined as “an individual’s perception of their position in life in the context of the culture and value systems in which they live and in relation to their goals, expectations, standards and concerns” [5].

In the context of chronic diseases, it has been observed that their slow progression, long duration, the presence of multiple symptoms, and the need for constant drug treatments and therapies significantly affect the quality of life, which tends to decline alongside the functional deterioration of the affected individual [6,7]. Approximately 75% of patients with heart failure report difficulty in performing activities of daily living, making the support of both informal caregivers (such as family members or friends) and formal caregivers (such as paid professionals) essential [8]. In this setting, the role of the nurse is pivotal, providing not only medical care but also essential emotional and educational support to both patients and caregivers, thereby enhancing the overall management of the disease and improving quality of life outcomes [9].

The figure of the family caregiver differs from that of the formal caregiver, who does not have a personal relationship with the caregiver and performs their role for financial compensation [10]. Carrying out the role of a caregiver for patients with heart failure has negative physical, psychological, social, and spiritual consequences, exposing the caregiver to a higher risk of depression and a lower perceived quality of life compared to the general population [11]. The quality of life of the patient and that of their caregiver affect one other; increased stressful conditions and mental and physical distress in the caregiver are directly associated with a worsening of the patient’s symptoms and health outcomes, increased re-hospitalizations, and mortality, highlighting the importance of monitoring this parameter in caregivers [12]. Nurses play a critical role in this dynamic, as they are often the primary point of contact in monitoring the health and well-being of both patients and caregivers, providing necessary interventions and support systems to mitigate these risks [13]. Among the instruments used to measure the quality of life, two types are identified: generic instruments (among which SF-36 is the most widely used), developed for assessment in the general population, and specific instruments, developed in order to assess specific contexts and diseases [14]. While valid for making global assessments and comparisons across different populations, generic tools are less responsive and sensitive than disease-specific tools; even in the specific context of heart failure, the need for specific tools for assessing quality of life in the patient and caregiver is highlighted [15,16].

To date, there are two questionnaires, validated in Italian, for assessing quality of life in the caregiver caring for a patient with heart failure: the Dutch Objective Burden Inventory (DOBI) and the Caregiver Burden Questionnaire for Heart Failure (CBQ-HF) [15,17].

Both questionnaires have a good level of reliability and validity, but they do not probe an important aspect of quality of life, the spiritual one. A recent systematic review in 2020 [18] highlighted the presence of spiritual distress in patients with heart failure and their caregivers, identifying the importance of identifying and treating it in a comprehensive multidisciplinary view. The perception of increased spiritual well-being in the studies analyzed by the review, both by the patient and caregiver, correlated with an improved overall quality of life [18]. Nurses, with their holistic approach to care, are essential in addressing these spiritual needs, integrating this aspect into the broader care plan to support comprehensive well-being [19]. To this end, an instrument, the FAMQOL (Family Caregiver Quality of Life), was developed with excellent reliability and validity characteristics. The questionnaires consists of 16 items and is the only one through which caregivers can express the impact of caregiving on all the previously mentioned aspects of well-being: physical, psychological, social, and spiritual [20]. To date, the instrument has been translated and validated in Turkish and Portuguese but there is no version of it available in Italian [21,22]. 

This study aims to translate and culturally adapt the FAMQOL from English to Italian, enhancing its utility in nursing research and practice to better identify and support caregiver well-being.

## 2. Materials and Methods

### 2.1. Study Design

A study of the linguistic validation and cultural adaptation of the FAMQOL scale was conducted by applying the guidelines dictated by the European Organization for Research and Treatment of Cancer (EORTC) in 2017 [23].

Before the translations were carried out and the data collected, formal permission was requested from the author of the original instrument. The ethics committee of the Humanitas Clinical Institute Rozzano, Milan, Italy, approved the trial by ruling no. 3684, dated 29 November 2023.

### 2.2. Participants, Setting, and Criteria

Before participating in this study, caregivers were informed and provided their authorization by signing an informed consent form. This study included caregivers who met the following criteria: caregivers of patients with heart failure receiving care at the Heart Failure Cardiology Unit and the Heart Failure Clinic of the Humanitas Research Hospital in Rozzano (MI); caregivers who perform at least two activities at home as identified by the Oberst Caregiving Burden Scale; and caregivers fluent in Italian, both spoken and written. Caregivers who provided care in exchange for financial compensation (formal caregivers) or who did not provide consent to participate were excluded from this study. 

### 2.3. Family Caregiver Quality of Life Scale

The original instrument was developed in the U.S. context, but it does not possess distinctive cultural characteristics that would invalidate its translation into Italian. Structured in the form of a questionnaire, the FAMQOL consists of 16 questions with 5-option Likert responses, ranging from “Strongly Disagree” to “Strongly Agree.” The questions are placed within four domains—physical, psychological, social, and spiritual—as follows: for physical well-being, items 1-5-8-9; for psychological well-being, items 2-3-4-6; for social well-being, items 7-10-11; and for spiritual well-being, items 12-13-14-15-16. The total score of each subscale can be from 4 to 20, and the total score can be from 16 to 80. Higher scores are indicative of a better QoL. None of the items are reverse-coded or negatively worded. The FAMQOL, which is a short and easily applied questionnaire, was self-administered electronically and could also be administered via telephone or filled out by the participants themselves (Supplementary File).

### 2.4. Linguistic Validation

For methodological purposes, the EORTC (2017) guidelines were used. As a result of the translation process, the guidelines report, “the final version should be linguistically and conceptually correct, understandable, culturally acceptable and non-offensive, and reflect the wording and structure of the source version” [23]. The guidelines provide 5 stages: (1) forward translations; (2) reconciliation; (3) back translations; (4) back translations’ report and reconciliation; and (5) pilot testing.

Following the author’s receipt of the original English language instrument, it was sent to two translators, who independently carried out the translation of the scale from English to Italian (forward translation). Both translators are native Italian speakers and fluent in English, holding Master’s degrees in English Language and Literature and Specialist Translation and Conference Interpreting in English. Following the production of the two translations, a meeting was held between the Study Coordinator and the two translators in order to generate a single Italian translation (reconciliation). According to the EORTC guidelines, Option 8 of the Reconciliation Protocol was applied: “To make a new translation out of the two with some modifications/additions, adapting translation B to A.”. The resulting version was sent to two other translators, who performed back translation from Italian to English, also holding Master’s degrees in English Language and Literature and Specialized Translation and Conference Interpreting in English. Both translators are native Italian speakers. All translations performed were then discussed by the Translation Unit, which was established as an expert group including 1 Nurse Expert in the field of heart failure and 2 Nurse Experts in the field of clinical research, all of whom spoke English fluently. Through the Translation Unit’s discussion, a single Italian translation was produced for testing. Before the pilot test was performed, the resulting translations were sent to the author of the original instrument for the purpose of further approval.

### 2.5. Pilot Testing

Pilot testing was performed for the purpose of assessing the comprehensibility of the questionnaire through its administration. The FAMQOL questionnaire translated into Italian was administered at the heart disease department and heart failure outpatient clinic of the Humanitas Research Hospital in Rozzano (MI).

The questionnaire was administered in a study population (monolingual Italian) of at least 15 caregivers meeting inclusion and exclusion criteria, after completion of informed consent. These criteria were derived from the original study [20]. In case of exclusion or withdrawal of a caregiver from the study, another caregiver could be enrolled as a replacement. Caregivers of patients with heart failure followed at the decompensation cardiology department and the heart failure outpatient clinic of the Humanitas Research Hospital in Rozzano (MI), who perform at home at least 2 activities identified in the Oberst Caregiving Burden Scale and are fluent in Italian language, both spoken and written, were included in the study. In contrast, caregivers practicing the role for financial compensation (formal caregivers) were excluded from the study.

Pilot testing took place in two stages: (1) administration of the questionnaire to the study population; (2) individual caregiver interview conducted by the Study Coordinator to identify any: response difficulties, comprehension difficulties, confounding factors, and disturbing factors. The questionnaire was administered in paper format for the only purpose of allowing the caregiver to assess its comprehensibility. Such questionnaires were not retained. A report was produced for each interview conducted, as directed by the EORTC containing information regarding the inclusion/exclusion criteria and caregivers’ comments regarding the questionnaire items. These reports were collected anonymously, in paper format and stored in sealed envelopes. A random 6-digit numerical code was generated for each report through the use of Blia software (https://www.blia.it, accessed on 28 November 2023) for the sole purpose of distinguishing them from others and in no way traceable to the respondent. Finally, all observations made by the study population were summarized for each individual item. These observations were discussed together with the Translation Unit (Supplementary File).

## 3. Results

Two independent translators, both native Italian speakers and fluent in English, carried out the forward translations. The translations were then reconciled into a single Italian version through a coordinated discussion. This version was then submitted to two other translators for back translation into English. All translations were reviewed by a group of experts, consisting of one nurse with specialized expertise in heart failure and two clinical research experts. The nurse is a certified specialist with extensive experience in managing patients with heart failure, and the clinical research experts hold advanced degrees in their respective fields, with significant experience in clinical studies and translation accuracy. This team ensured that the final version was linguistically and conceptually correct, comprehensible, and culturally acceptable. Their combined expertise provided a thorough review to address both the clinical relevance and the linguistic quality of the translations. The pilot test was conducted at the department of heart diseases and the heart failure outpatient clinic of the Humanitas Research Hospital in Rozzano. The translated FAMQOL questionnaire was administered to a sample of 15 caregivers, selected based on inclusion and exclusion criteria derived from the original study. During the pilot test, caregivers completed the questionnaire and participated in individual interviews to identify any difficulties in comprehension or response. The observations collected were used to make further improvements to the translation (Table 1).

The obtained pre-test translation was administered to 16 caregivers. Fifteen caregivers were recruited because they met the inclusion criteria. One caregiver enrolled for the interviews was excluded from the study because he performed the role for financial compensation. During completion of the form, a report consisting of two sections was prepared: a first part aimed at identifying inclusion and exclusion criteria and a second part aimed at collecting observations on the items from the recruited subjects (extrapolated from the EORTC 2017 protocol) (Table 2).

Each subject, following completion of the questionnaire, was asked for verbal feedback regarding each item. Four characteristics were probed: difficult, confusing, offensive, and use of difficult words. Of the 15 subjects interviewed, 14 informed the interviewer that they did not identify any of the characteristics for all the items on the questionnaire and did not report any observations. “No Observations” was then included within the reports. In contrast, the interview report code 723,805 identified the introduction to questions 1–3 and 8–12 as “difficult” and “confusing.” The interviewer reported the recruited subject’s comments: “The term “Caregiver” could be confusing and difficult to understand for individuals with limited English proficiency”. A discussion was held with the interviewee for the identification of a possible alternative to the term, but failed to identify it. 

The observation was submitted to the Translation Unit and the translators of the scale: it was decided to keep the term “Caregiver” as it is now in common use in the Italian language; moreover, the observation was carried out on an introductory component and not on the items of the scale itself.

## 4. Discussion

There is strong evidence to date that caregivers of patients with heart failure, as a consequence of their responsibility, experience issues related to physical, psychological, emotional, and spiritual well-being that result in a reduced quality of life [11]. The evolution of the pathology over time often results in a worsening of the caregiver’s perceived quality of life, resulting in decreased care support for the patient, increased complication rates, and re-hospitalizations of the patient [12,24,25]. It has been observed that the use of appropriate tools to monitor quality of life in the caregiver has allowed the early identification of impairment, enabling early intervention [26]. The possibility of the early identification of impairment in quality of life also allows targeting different specific interventions, such as psycho-educational interventions, leading to significant improvements not only in the caregiver’s perceived well-being, but also in the caregiver’s burden, depression, and knowledge of heart failure [27].

Moreover, recent studies have highlighted the importance of continuous assessment and intervention strategies tailored to the evolving needs of caregivers over time [28,29]. This dynamic approach ensures that the interventions remain relevant and effective, addressing the changing challenges faced by caregivers as the patient’s condition progresses. Additionally, integrating these tools into routine clinical practice has been shown to enhance the overall management of chronic conditions like heart failure by fostering a collaborative environment where both patients and caregivers feel supported and empowered [30].

The role of nurses in this context is paramount. Nurses are in a unique position to utilize these tools effectively, given their frequent and direct contact with both patients and caregivers. They can play a critical role in the early identification of quality of life impairments and the implementation of targeted interventions. Through regular assessments and interactions, nurses can provide essential support, education, and resources to caregivers, thereby improving both patient and caregiver outcomes. This holistic approach ensures that the physical, psychological, social, and spiritual needs of caregivers are addressed comprehensively [31]. Furthermore, the involvement of nurses in caregiver education has been shown to significantly reduce the emotional burden on caregivers by improving their confidence and competence in managing the patient’s condition [32]. This, in turn, leads to better patient adherence to treatment plans and a reduction in adverse events, thereby improving the overall quality of care provided [33]. 

The linguistic validation and cultural adaptation of the Family Caregiver Quality of Life scale has made it available in Italian, enabling its use by caregivers in all caregiving settings. Its conciseness and ease of use make the FAMQOL a useful tool for the ongoing monitoring of caregivers’ quality of life, while its comprehensiveness in investigating all four aspects of well-being make it a questionnaire capable of targeting caregiver interventions in specific areas of caregiving [5,7,20]. This adaptability is crucial in ensuring that the tool remains relevant across diverse caregiving environments, particularly in a multicultural context like Italy, where regional differences might impact the caregiving experience. By providing a reliable means of assessing caregiver quality of life, the FAMQOL serves as a foundation for developing personalized care plans that address the unique challenges faced by caregivers in different settings [20].

Nurses, with their extensive training and holistic approach to patient care, are ideally suited to administer such tools and interpret their results. They can offer tailored interventions that address the specific needs identified through the FAMQOL, thereby enhancing the overall support system for caregivers [34]. Additionally, the continuous education provided by nurses helps caregivers better understand heart failure management, reducing anxiety and improving care techniques [35]. This continuous education is not only beneficial for the immediate caregiving tasks but also contributes to long-term caregiver resilience, enabling them to better cope with the ongoing demands of caregiving and reducing the risk of burnout [36].

The use of the EORTC guidelines allowed for a translation of the instrument, testing its comprehensibility on a study population. Collaboration with experienced English-language translators allowed for careful cross-cultural adaptation, evaluating among equivalent terms those that were simpler and clearer for the general Italian population. The back translation phase and the exchange of information with the author of the original tool ensured semantic equivalence between the original document in English and the tool generated in Italian. This rigorous process underscores the importance of maintaining the integrity of the original instrument while ensuring that it is culturally and linguistically appropriate for the target population.

Ultimately, the tool translated into Italian was found to be understandable by the caregivers of patients diagnosed with heart failure. However, it is important to note that cultural adaptation is just the initial step in the validation process of a questionnaire. Therefore, additional studies are necessary to evaluate whether the instrument retains the same psychometric characteristics as the original version [37]. Future research should focus on further validating the tool’s reliability and validity in the Italian context, ensuring that it accurately reflects the experiences and challenges faced by Italian caregivers. Such validation is essential for the tool to be widely adopted in clinical practice and for it to effectively guide interventions aimed at improving caregiver quality of life.

### Study Limitations

One limitation of this study is that it primarily focused on the linguistic and cultural adaptation of the FAMQOL scale, without extensive validation of its psychometric properties in the Italian context. Further research is needed to confirm the reliability and validity of the Italian version of the questionnaire. Additionally, while the study’s sample size was relatively small, a more significant limitation might be the lack of information on the homogeneity or heterogeneity of the participants. This could impact the generalizability of the findings, as the sample may not fully represent the diverse characteristics of the broader caregiver population. Future studies should aim to include a larger and more diverse population to better understand the questionnaire’s applicability across different caregiver demographics.

## 5. Conclusions

The adaptation and validation of the FAMQOL scale in Italian represent significant progress in supporting the caregivers of patients with heart failure. Nurses, with their role as caregivers and their surrounding environment, are key to the effective use of this tool, ensuring comprehensive support for healthcare professionals. The integration of this tool into routine nursing practice not only enhances the support system for caregivers but also contributes to a more holistic approach to patient care. By systematically monitoring the quality of life of caregivers, nurses can identify and address potential issues before they escalate, thereby improving both caregiver well-being and patient outcomes. Early findings suggest that the Italian version of the FAMQOL is understandable and potentially useful in care environments. Nevertheless, it is crucial to conduct further research to assess the psychometric properties of the Italian version, ensuring that it maintains the reliability and validity of the original instrument. Additionally, future studies should investigate the long-term effects of using the FAMQOL on both caregiver burden and patient health outcomes, thereby providing a more comprehensive understanding of its impact.

## Figures and Tables

**Table 1:** FAMQOL questionnaire’s translation and re-evaluation process.

Original Instrument	Forward Translation a	Forward Translation b	Reconciliation	Back Translations	Pre-Test Translation	Observations
As a caregiver,	In quanto caregiver,	Come caregiver,	In quanto caregiver,	As a caregiver,	In quanto caregiver,	*/*
D1: I seem to get sick more often.	Mi sembra di ammalarmi più frequentemente.	Mi sembra di sentirmi male più frequentemente.	Mi sembra di ammalarmi più frequentemente	I feel to get sick more often.	Mi sembra di ammalarmi più frequentemente	“Get sick” is correctly translated as “ammalarsi” rather than “sentirsi male.”
D2: I am overwhelmed.	Mi sento sopraffatto/a.	Sono sopraffatto.	Sono sopraffatto/a	I am overwhelmed.	Sono sopraffatto/a	“Sono sopraffatto” was used as it is more recurrent in common usage and is the literal translation of the English version.
D3: I feel selfish when considering my own needs.	Mi sento egoista a considerare i miei bisogni.	Mi sento egoista quando mi occupo dei miei bisogni.	Mi sento egoista a considerare i miei bisogni.	I feel selfish to consider my own needs.	Mi sento egoista a considerare i miei bisogni.	“Considerare” was used rather than “occuparsi” as it is the correct translation of “considering” and involves active action.
Because of caregiving,	Essendo un/una caregiver,	A causa del caregiving,	A causa del caregiving	Because of caregiving	A causa dell’assistenza alla persona	
D4: I am tired	Sono stanco/a.	Mi sento stanco.	Sono stanco/a	I am tired.	Sono stanco/a	\
D5: My physical health has suffered.	La mia salute fisica ne ha risentito.	D5 La mia salute fisica ne risente.	La mia salute fisica ne ha risentito.	My physical health has suffered	La mia salute fisica ne ha risentito	\
D6: I am strained emotionally.	Sono emotivamente esausto/a	Mi sento emotivamente provato	Mi sento emotivamente provato/a.	I feel emotionally drained.	Mi sento emotivamente provato/a.	It was decided to use “provato” because “esausto” is an adjective having a greater degree of expressive intensity than “strained.”
D7: I am socially isolated.	Sono socialmente isolato/a.	Mi sento isolato socialmente.	Sono socialmente isolato/a.	I am socially isolated.	Sono socialmente isolato/a.	\
Even though I am a caregiver,	Nonostante io sia un caregiver,	Nonostante io sia un caregiver,	Nonostante io sia un caregiver	Although I am a caregiver,	Nonostante io sia un/una caregiver	\
D8: I am still able to exercise like I want.	Sono ancora in grado di fare l’attività fisica che desidero.	Sono ancora grado di fare esercizio come voglio	Sono ancora in grado di fare l’attività fisica che desidero.	I am still able to do the physical activity as I want.	Sono ancora in grado di fare l’attività fisica che desidero.	\
D9: I am able to get to my own checkups with doctors, dentists, and other healthcare providers.	Sono in grado di fare controlli periodici con dottore, dentista e altri professionisti sanitari.	Ho modo di fare i miei controlli con medici, dentisti e assistenti sanitari.	Ho modo di fare i miei controlli periodici con medici, dentisti e altri professionisti sanitari	I can have my regular checks with doctors, dentists, and other healthcare providers.	Ho modo di fare i miei controlli periodici con medici, dentisti e altri professionisti sanitari	The two versions have been merged because version A reports professionals in the singular and version B reports “controlli” generically.
D10: I am able to participate in enjoyable activities.	Posso prendere parte ad attività piacevoli.	Sono in grado di partecipare ad attività ludiche.	riesco a prendermi momenti di svago	I am able to take part in leisure activities.	riesco a prendermi momenti di svago	“Prendermi” was used because it includes individual activities, as opposed to “partecipare”. “Momenti di svago” was used because it was intended as a time of detachment and not an activity aimed at having fun.
D11: I am able to maintain personal relationships with others.	Riesco a mantenere relazioni interpersonali con terzi.	Sono in grado di mantenere relazioni interpersonali.	Riesco a mantenere relazioni interpersonali con altri	I am able to maintain interpersonal relationships with others.	Riesco a mantenere relazioni interpersonali con altri	From version A, a modification was applied by changing “terzi” to “altri” as it is less formal.
D12: I am able to practice religious activities if I want to.	Posso partecipare, se lo voglio, ad attività religiose.	Sono in grado di professare attività religiose, se lo desidero.	Posso partecipare, se lo voglio, ad attività religiose	I can take part in religious activities if I want to.	Posso partecipare, se lo voglio, ad attività religiose	\
Caregiving…	Assistere…	Il caregiving…	Il caregiving…	Caregiving…	I’ assistere la persona…	\
D13: Adds to my purpose or mission in life.	È un valore aggiunto per la mia vita o per i miei obiettivi.	Dà valore al mio scopo e missione di vita.	Dà valore al mio scopo o missione di vita	Adds value to my purpose or mission in life.	Dà valore al mio scopo o missione di vita	\
D14: Adds to my feelings of inner strength.	Aumenta la mia sensazione di forza interiore.	Contribuisce ad alimentare la mia forza interiore.	Aumenta la mia sensazione di forza interiore.	Increases my feeling of interior strength.	Aumenta la mia sensazione di forza interiore.	\
D15: Gives me a sense of inner peace	Mi dà un senso di pace interiore.	Mi dà un senso di pace interiore.	Mi dà un senso di pace interiore	Gives me a sense of inner peace	Mi dà un senso di pace interiore	\
D16: Gives meaning to my life.	Dà senso alla mia vita.	Dà significato alla mia vita.	Dà senso alla mia vita.	Gives meaning to my life.	Dà senso alla mia vita.	\

**Table 2:** Inclusion and exclusion criteria.

ID Report	Fluent in Italian	Formal Caregiver	Caregiver Activities *
698,425	YES	NO	12/15
931,009	YES	NO	10/15
181,931	YES	NO	7/15
153,113	YES	NO	8/15
322,898	YES	YES	12/15
227,318	YES	NO	4/15
210,728	YES	NO	13/15
827,962	YES	NO	8/15
110,031	YES	NO	10/15
177,653	YES	NO	12/15
723,805	YES	NO	13/15
389,946	YES	NO	3/15
917,494	YES	NO	9/15
109,421	YES	NO	5/15
247,580	YES	NO	11/15
633,209	YES	NO	10/15

Legend: * activities at home declared by the caregivers as identified by the Oberst Caregiving Burden Scale.

## Data Availability

Data availability statements are available upon request from the corresponding author of this study.

## Supplementary Materials

The following supporting information can be downloaded at https://www.mdpi.com/article/10.3390/nursrep14030171/s1, Study questionnaire, Italian and English version; and EORTC Checklist.

## Public Involvement Statement

There was no public involvement in any aspect of this research.

## Guidelines and Standards Statement

This manuscript was drafted against the COnsensus-based Standards for the selection of health Measurement INstruments (COSMIN) guidelines for reporting the psychometric validation of measurement instruments [38].
