# Supplementary material for: Language Validation and Cultural Adaptation of the Italian Version of the Family Caregiver Quality of Life Scale
Source: Nurs Rep. 2024 Sep 6;14(3):2302–12. doi: 10.3390/nursrep14030171 (PMC11417854; doi:10.3390/nursrep14030171)
Supplement: Supplementary file 1 [file nursrep-14-00171-s001.zip › nursrep-3138571-supplementary.pdf]

SUPPLEMENTARY FILES

FAMQOL English Version (original)

This group of questions is about how providing care to the heart failure patient has affected your life. Circle the number that most closely represents your level of agreement.

	Strongly Disagree	Disagree	Neither Disagree or Agree	Agree	Strongly Agree
As a caregiver,					
1. I seem to get sick more often.	1	2	3	4	5
2. I am overwhelmed.	1	2	3	4	5
3. I feel selfish when considering my own needs.	1	2	3	4	5
Because of caregiving,					
4. I am tired.	1	2	3	4	5
5. My physical health has suffered.	1	2	3	4	5
6. I am strained emotionally.	1	2	3	4	5
7. I am socially isolated.	1	2	3	4	5
Even though I am a caregiver,					
8. I am still able to exercise like I want.	1	2	3	4	5
9. I am able to get to my own checkups with doctors, dentists, and other health care providers.	1	2	3	4	5
10. I am able to participate in enjoyable activities.	1	2	3	4	5
11. I am able to maintain personal relationships with others.	1	2	3	4	5
12. I am able to practice religious activities if I want to.	1	2	3	4	5
Caregiving...					
13. Adds to my purpose or mission in life.	1	2	3	4	5
14. Adds to my feelings of inner strength.	1	2	3	4	5
15. Gives me a sense of inner peace.	1	2	3	4	5
16. Gives meaning to my life.	1	2	3	4	5

FAMQOL Italian Version

Questo insieme di domande riguarda le conseguenze che l'assistere un paziente affetto da scompenso cardiaco ha avuto sulla tua vita. Cerchia il numero che meglio rappresenta il tuo livello di consenso.

In quanto caregiver,	In forte disaccordo	In disaccordo	Né in accordo né in disaccordo	D'accordo	Molto d'accordo
1. Mi sembra di ammalarmi più frequentemente.	1	2	3	4	5
2. Sono sopraffatto/a.	1	2	3	4	5
3. Mi sento egoista a considerare i miei bisogni.	1	2	3	4	5
A causa dell'assistenza alla persona,					
4. Sono stanco/a.	1	2	3	4	5
5. La mia salute fisica ne ha risentito.	1	2	3	4	5
6. Mi sento emotivamente provato/a.	1	2	3	4	5
7. Sono socialmente isolato/a.	1	2	3	4	5
Nonostante io sia un/una caregiver,					
8. Sono ancora in grado di fare l'attività fisica che desidero.	1	2	3	4	5
9. Ho modo di fare i miei controlli periodici con medici, dentisti e altri professionisti sanitari.	1	2	3	4	5
10. riesco a prendermi momenti di svago.	1	2	3	4	5
11. Riesco a mantenere relazioni interpersonali con altri.	1	2	3	4	5
12. Posso partecipare, se lo voglio, ad attività religiose.	1	2	3	4	5
L' assistere la persona,					
13. Dà valore al mio scopo o missione di vita.	1	2	3	4	5
14. Aumenta la mia sensazione di forza interiore.	1	2	3	4	5
15. Mi dà un senso di pace interiore.	1	2	3	4	5
16. Dà senso alla mia vita.	1	2	3	4	5

EORTC Translation Flowchart

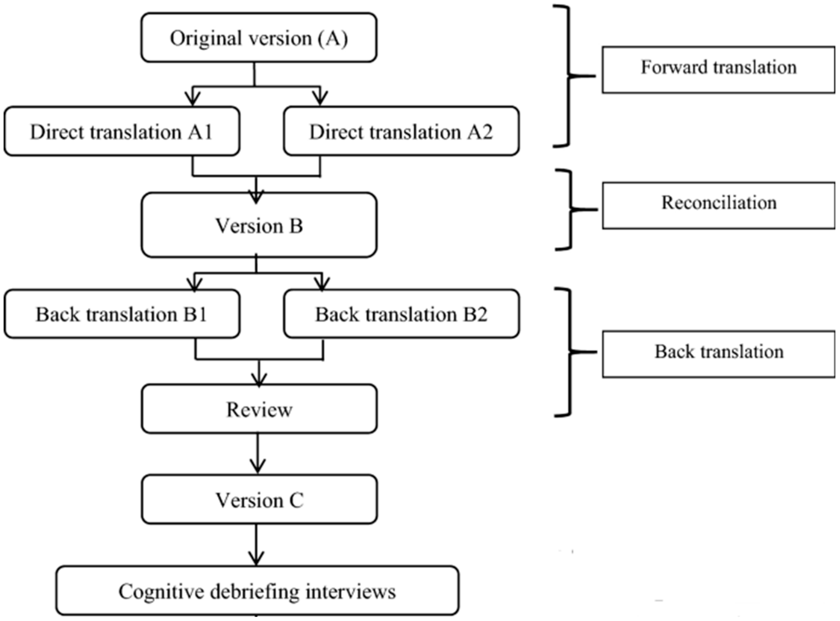

Quality-of-Life Group Translation Procedure". European Organisation for Research and Treatment of Cancer (EORTC),2017. (Kuliś et al. 2017)
